# Provision of care for people with HIV migrating from Ukraine: preparing for a long-term response

**DOI:** 10.1097/QAD.0000000000004147

**Published:** 2025-04-03

**Authors:** Miłosz Parczewski, Christoph Boesecke, Pavel Khaykin, David Jilich, Raluca Pătraşcu, Justyna Kowalska, Iurie Climasevschi, Teymur Noori

**Affiliations:** a Department of Infectious, Tropical Diseases and Immune Deficiency, Pomeranian Medical University in Szczecin, Szczecin, Poland; b Department of Medicine I, Bonn University Hospital, Bonn; c Practice MainFachArzt, Frankfurt, Germany; d Faculty Hospital Bulovka and 1st Faculty of Medicine, Department of Infectious Diseases Charles University, Prague, Czech Republic; e National Institute for Infectious Diseases ‘Prof dr Matei Bals’, University of Medicine and Pharmacy Carol Davila, Bucharest, Romania; f Department of Adults’ Infectious Diseases, Medical University of Warsaw; g Hospital for Infectious Diseases in Warsaw, Warsaw, Poland; h Coordination Unit of the National Programme for Prevention and Control of HIV/AIDS and STIs, Hospital for Infectious Diseases ‘Toma Ciorba’, Chisinau, Rep. of Moldova; i European Centre for Disease Prevention and Control (ECDC), Stockholm, Sweden.

**Keywords:** Antiretroviral treatment, HIV migrant care, refuge, standards of care, Ukraine

## Abstract

Russia's invasion and war in Ukraine have caused a major humanitarian crisis among Ukrainian citizens, but also specifically affected diagnosis and provision of HIV care. As Ukraine remains the country with the second highest (after Russia) HIV incidence in Europe, the forced migration resulting from the war has required urgent and targeted responses to allow for uninterrupted access to medical care and antiretroviral drug supply in neighboring countries and beyond. Response and integration of people with HIV (PWH) has been swift across European countries, but several challenges remain. Key challenges relate to the expansion of unstigmatized HIV testing to tackle late diagnoses, development of legal frameworks allowing for access to medication not registered or under patent protection in other European countries, diagnosis and treatment of key comorbidities including tuberculosis (TB) and hepatitis C virus (HCV), vaccination programs, and continued surveillance for drug resistance and changes in molecular epidemiology.

## Introduction on overall migration crisis related to war in Ukraine

Russia's invasion and war in Ukraine has resulted, and continues to result, in notable humanitarian consequences with more than 6.4 million people seeking refuge globally, of these the majority (5.9 million) in Europe and 4.3 million provided with temporary protection status in the European Union/European Economic Area (EU/EEA) countries [1]. The EU/EEA responded in part by implementing the Temporary Protection Directive allowing Ukrainian refugees easier access to residency and employment as well as healthcare services [2]. Within this framework, several countries in the EU/EEA received and provided national protection to a large number of Ukrainian refugees with 1.9 million, 1.2 million, and 0.63 million refugees received by Poland, Germany, and Czech Republic, respectively, as of the end of 2024 [1]. As all countries continue to ensure international standards for protection of rights and services including access to medical services, here, we focus on the provision of high-quality standards of HIV care to Ukrainian refugees living in neighboring European countries.

Ukraine carries the second to highest burden of HIV infection in Europe with an estimated HIV prevalence of 0.66–1.0% (cumulative total 348 895 people with HIV (PWH) and 37.1 new diagnoses/100 000 population in 2023. An estimated 25% of all people with HIV are not aware of their status [3].

Russia's war in Ukraine has recently entered its third year, and as a consequence, it is important that hosting countries plan for a long-term strategy in terms of providing early diagnosis and high-quality HIV care for Ukrainian refugees with HIV. Interestingly, as of May 2023, a total of 6519 refugees were reported to receive antiretroviral therapy (ART) in Europe, which is lower than the predicted estimate [4], which may reflect unreported but also untreated cases and calls to continued improved access to healthcare among Ukrainian refugees. For this reason, this narrative review provides an overview of the key challenges and future directions, as it pertains to early diagnosis and the provision of high-quality standards of care for Ukrainian PWH in neighboring countries. For this purpose, we searched the medical databases for studies and data relevant for migration and refugee care from Ukraine. Hand searching from the retrieved articles was performed for key information for each country. We included data from relevant articles published in English, Ukrainian and Russian and supplement it with personal communications with treatment centres and Ukrainian refugees in care.

The recommendations suggested in this perspective were formulated based on the three pillars: a nonsystematic literature review related to the topic; expert opinion and: a review of challenges reported by key countries experiencing war related migration (see basis for country selection below). Experts selected for this review were selected based on the following criteria: expertise in HIV medicine with notable track record and engagement in care of migrant populations.

## Disruption of HIV care in Ukraine following outbreak of war

Russia's invasion and consequent war in Ukraine on 27th of February 2022 resulted in significant disruptions of medical care provision with more than 1500 attacks on and damage of 630 healthcare facilities in Ukraine, affecting also HIV medical care [5]. Despite these difficulties, efforts to control the HIV epidemic in Ukraine remain extensive with approximately 80% of HIV centres functioning and only a small decline in the number of patients on ART (> 130 000 pre-war, as of February 2022 and 118 345 at the end of 2023) [6]. The majority of treated PWH receive the WHO-recommended first-line regimen [7], tenofovir disoproxil/lamivudine/dolutegravir (TLD – not available in European Union countries as single tablet regimen due to patent protection) allowing to maintain at least 95% viral suppression among treated individuals. Data on the HIV care continuum are missing for the temporarily occupied parts of Eastern Ukraine, including Donetsk, Luhansk, Zaporizhzhia, Kherson regions, AR Crimea, and the city of Sevastopol, where the HIV prevalence prewar was high. It should be noted that HIV transmission among people who inject drugs (PWID) in Ukraine is on the rise, amounting to 38% of the total newly diagnosed cases in 2022. The specific reasons for this expansion include intertwining of injection drug use and unsafe sexual practices, namely condomless sex [8]. The number of people on opioid agonist therapy (OAT) and receiving HIV pre-exposure prophylaxis (PrEP) continue to rise considerably (38 and 130%, for 2022 and 2023, respectively) [5]. There are scarce data on the distribution and transmission of HIV drug resistance. The A6 subtype is predominant in Ukraine and was previously associated with decreased virologic efficacy among patients receiving long-acting injectables (LAI) with cabotegravir/rilpivirine (CAB/RPV) [9–11]. Internal migration occurring since first Russian aggression acts starting in 2014 has been described as affecting the HIV transmission along with new resistance patterns [12]. Migration from Ukraine is already notably affecting the HIV subtype variability across Europe with increasing frequency of A6 variant reported [9,13].

## Impact of war in Ukraine on HIV and comorbidities epidemiology in host countries

Overall displacement of people from Ukraine has influenced the epidemiology of HIV in Europe, increasing the number of previously positive HIV diagnoses and new HIV diagnoses especially in female populations, and also notably influencing numbers of perinatal transmissions [14,15]. There is also a notable change in molecular epidemiology of HIV in Europe with influx of A6 variants and expansion of transmission networks related to this viral subtype with molecular data showing that war-related influx of displaced people fuels the epidemic [13,16]. In 2022, 10.2% of all HIV diagnoses in the EU/EEA was recorded among Ukrainian refugees resulting in a 10-fold increase compared to 2021 [15]. Importantly, 9.3% of these were coded as new HIV diagnoses, the majority (61%) were women with predominant heterosexual transmission mode (76.6%), followed by IDU (10.2%) among people with known transmission category. The proportion of late diagnosis (with lymphocyte count <350 cells/μl or AIDS-defining condition) at care entry [17] among newly identified cases of HIV infection reached 47.0%. Although the majority (∼90%) of refugees from Ukraine were already aware of their status, the numbers of late diagnosed individuals are increasing [18]. Influx of children with HIV also poses a notable challenge for the provision of care among people from Ukraine, especially in the central European countries due to increasing numbers of pediatric populations requiring care not only from the perspective of antiretroviral management but also diagnosis and treatment of coinfections, including TB. A further challenge relates to the fact that a high proportion of orphans from parents who have died from HIV are in need of specialized psychological support, while resources in relation to psychological support in the native language are limited [19].

TB remains a key comorbidity among PWH from Ukraine. Already in 2022, following the war in Ukraine, the number of notified TB cases among Ukrainian citizens across European countries increased fourfold [20]. In the prewar period, this population was associated with 0.5% of total number of notified TB cases (years 2019–2021) and this increased to 2.2% for 2022 in the EU/EEA. Among TB-diagnosed populations migrating from Ukraine, drug-resistant strains were reported in 31.9% of cases [21]. Also, the frequency of TB infection among the general population of Ukrainian refugees is considerable, with 13% of the sampled population having a positive interferon gamma release test result (IGRA-TB) in the recent study from Germany [22]. Furthermore, migration from Ukraine notably affects TB epidemiology in other European countries accepting large number of refugees such as in the Czech Republic or Slovakia [23]. Also, in these countries, rising numbers of drug resistant strains were noted with confirmation of transmission clusters among patients migrating from divergent regions of Ukraine and the possibility of recent transmissions outside the host country or during refuge but not in the host country.

Furthermore, rates of HCV and HBV infections are also expected to increase with anti-HCV antibodies observed in 3.6% of refugees [22]. Hepatitis B immunization rates among refugees from Ukraine are low, below 20% in adults, both for general refugee populations and PWH which call for a targeted immunization strategy [14,22].

The war had a significant impact on the social, emotional wellbeing of participants with common concerns on medication access and fears associated with war which negatively impacted adherence [24]. Refugees from Ukraine experience higher risk of psychological distress including stress-related mental health disorders, such as depression and anxiety often linked to social factors such as perceived insufficient support received in the host country [25,26].

## System preparedness and frameworks for optimal cascade of HIV care for refugee populations

Forced migration and refuge from Ukraine has affected public assistance, policy, and health systems in other European countries [27]. Countries providing immediate HIV care upon arrival of a person with HIV should consider a person's intention to settle in the host country or account for future migration plans to other host countries [28]. A protocol for medical data exchange was established in March 2022, at the request of the WHO Regional Office for Europe and in collaboration with multiple stakeholders, including the Ministry of Health of Ukraine, European AIDS Clinical Society, and ECDC (40). This protocol enables access to prior medical history of patients with HIV and takes into account legal regulatory requirements both of EU/EEA and Ukrainian law acts. The protocol has been tested on a sample of refugees arriving in HIV care in Poland showing a 98.7% linkage to medical data available in Public Health Centres of Ukraine [29].

After the onset of the war in Ukraine, a conceptual framework for sustaining the HIV continuum of care for displaced Ukrainian populations with HIV was proposed [30]. Within this framework, essential factors for optimization of care were outlined and subdivided into immediate, urgent, consolidation, and post war rebuilt phase. This framework should now be reviewed and updated from the perspective of observed epidemiological trends as described above.

In general, care provided to Ukrainian refugees with HIV has now entered the postacute “consolidation” phase where long-term solutions to testing and follow-up are needed, but several points from both immediate and urgent phases require continuous attention and optimization. Therefore, it should be emphasized that efforts related to the consolidation of care require combined management strategies including steps from all phases.

First, in the immediate phase provision of care, continuous access to ART as well as availability of medicines necessary for AIDS-related condition treatment, including drug-resistant TB, should be ensured. Also, continued efforts should be made to provide psychological support, maintain harm reduction support, and fight stigma.

Second, included in the urgent phase, access to drug resistance testing, both for newly diagnosed and virologically unsuppressed individuals is usually accessible across the existing laboratory networks in the host countries. Diagnostics and treatment of comorbidities with a special focus on women and underage populations, as well as appropriate infection prevention strategies, should be further reinforced, especially due to lower immunization rates in the Ukrainian refugee population compared to those in host countries [31]. It should be noted that many countries in the Central and Eastern European (CEE) region accepting refugees also struggle to introduce combination prevention (e.g., pre-exposure prophylaxis, which was available in Ukraine is often not available free of charge in the host country) [32].

## Country examples for provision of care

**Fig. 1:**
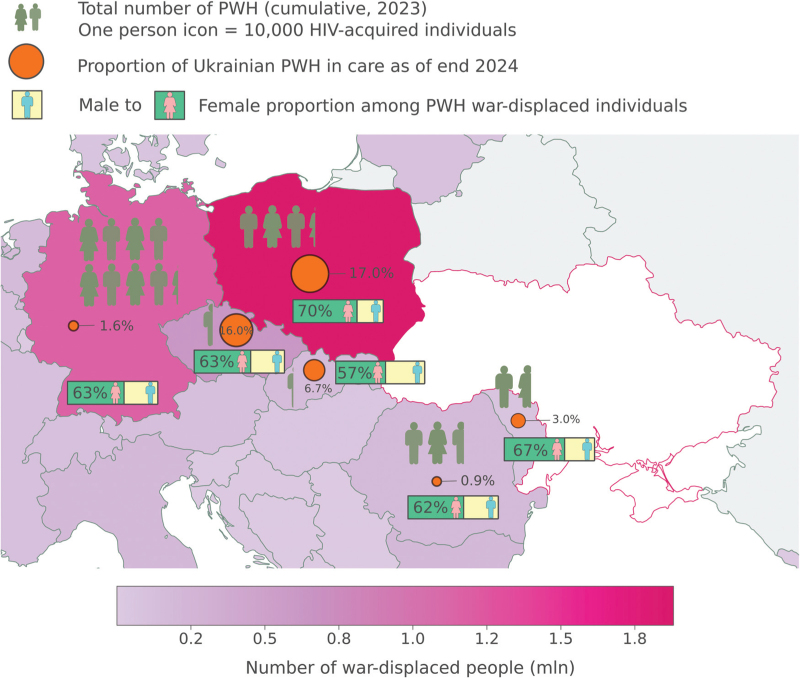
Map of selected countries with overview of overall war-related displacement from Ukraine, total number of people with HIV entering care since outbreak of war, proportion of migrants in care and female to male ratio.

To reflect key challenges in provision of HIV care for the Ukrainian refugees, we provide five country case studies of the challenges experienced: three from the countries with the highest number of registered refugees from Ukraine (Poland, Germany, and Czech Republic) and two further from countries with a large border with Ukraine, often being the first migratory place of refuge (Romania and Moldova). Overview of the country specific data is provided on Fig. 1 and Table 1.

**Table 1:** Summary characteristics of people with HIV migrating from Ukraine to selected countries from the context of host country epidemics.

Country	Total number of PWH (cumulative, 2023) [46]	Estimated total of PWH from Ukraine entering care since February 2022	Estimated proportion of Ukrainian PWH in care as of end 2024	Proportion of late diagnosed Ukrainian PWH entering care since February 2022	Proportion of women among Ukrainian PWH entering care since February 2022	Proportion of anti-HCV positive among Ukrainian PWH entering care since February 2022	Proportion history of tuberculosis Ukrainian PWH entering care since February 2022	Proportion of Ukrainian migrant PWH lost to follow-up since February 2022
Poland	32 918	3500	17.0%	69% [47]	70% [14]	28.7% [14]	10.1% [14]	10%
Germany	82 841	1400	1.6%	No data	63% [37]	12.2% [48]	3.5% [48]	No data
Czech Republic	5346	849	16.0%	71%	63%	23.4%	∼5–10%	∼ 5–10%
Republic of Moldova	17 008	289	3.0%	10%	67%	No data	0.01%	90%
Romania	28 008	171	0.9%	No data	62%	No data	6.4%	55.5%

Provided data relate to the PWH of migrating from Ukraine since February 2022 until the most recent data reported by countries (end of 2024). If no specific reference, data were obtained via personal communications with relevant national authorities and care centres. For the cumulative total number of HIV rates, most recent (2023 data) ECDC reports were used.

### Poland

Economic migration, as well as seeking refuge from Russian acts of aggression since 2014 was an ongoing process observed in Poland. As many other countries, Poland provided Ukrainian refugees with full access to the social system and adopted custom laws in order to enable massive migration [31]. Since HIV infections are eight times more prevalent in Ukraine compared to Poland [33], this has posed a significant challenge on the healthcare system, resulting in the integration of more than 3500 (including >100 children) new PWH into HIV healthcare services up until the end of 2023 and between an 18 and 20% increase in the number of treated patients. It must be emphasized that 89% of refugees were aware of their HIV status and 90% were on ART having undetectable (< 50 copies/ml) viral loads while only 5% entering care with viral load more than 1000 copies/ml. In the group entering care, 85.4% received integrase inhibitor treatment (74.3% on TLD) [14]. As TLD is not registered in the EU/EEA, there was a need to optimize and switch treatment regimen in 94.1% of patients.

The main mode of HIV transmission has as a result of the migration from Ukraine changed from MSM to an increasing number of heterosexual women posing challenges related to access to sex and culture sensitive care where addressing language barrier is of primary importance. One of the key continuous and unresolved challenges is related to the late diagnoses in individuals previously unaware of their HIV infection. Approximately two million refugees from Ukraine live in Poland (including both prewar refugee population and postwar refugees) and there is no information on the size of the HIV undiagnosed population. In fact, late diagnoses in this population exceeds 70% of new diagnosed cases; furthermore, in 40%, an opportunistic infection is diagnosed. Among the newly diagnosed population, 55% of PWH are women, but an increasing number of MSM, previously undisclosed for the fear of stigma, is also observed. Furthermore, 20% are diagnosed with replicating HCV (both groups) and 10% with syphilis [34]. Despite an urgent need, there are no targeted testing programs addressing this gap.

Cases of TB infection have been increasing in the general population due to migration, with doubled rates of disease among general population of foreigners between 2021 and 2022 [35]. TB is also the most common opportunistic infection in populations newly diagnosed with HIV, 25% being multidrug-resistant (MDR) or extensively drug resistant (XDR) and it is pivotal to maintain access to tuberculostatic drugs especially targeted against drug-resistant strains (bedaquiline, pretonamid, linesolid, moxifloxacin). A notable increase of TB infection among Ukrainian refugees with HIV has been observed [36].

Lastly, migration has resulted in the change of the HIV molecular epidemiological pattern in Poland with increasing numbers of observed infections with A6 variant introduced by migration but fueling MSM transmission clusters [9,16]. Transmitted drug resistance to rilpivirine reached 14% of cases, but integrase resistance was infrequently observed [14].

### Germany

At the end of 2023, there were around 1.14 million refugees in Germany seeking refuge from Ukraine, but it is impossible to estimate the exact numbers of people declaring long-term stay. Exact estimates of the number of war refugees PWH entering care from Ukraine are not available, but data from the Orange trial registered 941 patients with predominance of women (63.4%), 93% already on ART [71.4% on dolutegravir (DTG) based regimens], with 80.9% virologic suppression rates (HIV-1 viral load <50 copies/ml) and lymphocyte CD4^+^ cell count exceeding 500 cells/μl. Similarly to Poland, ART modification was frequent (72.9%), usually due to unavailability of previous antiretroviral combination [37]. The three most popular combinations for therapy switch were tenofovir disoproxil/emtricitabine (FTC/TDF)+DTG, tenofovir alafenamide/emtricitabine/bictegravir (B/FTC/TAF), or dolutegravir/lamivudine (DTG/3TC).

All Ukrainian refugees, including PWH, were provided with full access to health insurance identical to permanent German residents to ensure continued access to HIV care, but there is no specific targeted strategy for HIV testing of Ukrainian refugees at the national level. Of note, one-third of all HIV diagnoses still occur late (CD4^+^ T cell count < 350/μl and/or AIDS-defining illness) in the German general population reflecting substantial deficiencies in the national testing strategies [38]. As part of the mandatory HIV reporting in accordance with Infection Protection Law (IfSG), all new HIV diagnoses in Germany must be reported, but data with regard to Ukraine are not yet available. Information on known co-infections can also be submitted in the reporting. A co-infection with hepatitis C was reported in 12.2% of cases of Ukrainian origin, in 4.0% of cases with origin from other countries, and in 2.2% of cases of German origin. A known co-infection with TB was also reported more frequently in cases of Ukrainian origin (3.5%) than in cases originating from other countries (1.3%) or Germany (0.4%) [39]. TB treatment, HCV directly acting antiviral treatment as well as relevant HBV treatment is available within the full coverage of health insurance and for patients free of charge. In addition to language barriers and missing medical histories, the main challenge currently is the high number of people still being diagnosed late with HIV. While online translation tools have been of substantial help during consultations, initiatives so far to reduce the number of people being diagnosed late with HIV have still not succeeded, hence there are new endeavors to strengthen anonymous on-site testing at community checkpoints as well as promoting home-based self-testing for HIV.

### Czech Republic

All war refugees have full access to health insurance, including ART, provided for at least 6 months guaranteed by Czech Government on same level as Czech citizens. This period can be prolonged for persons in need. Despite lack of information about the exact total number of PWH from Ukraine entering the Czech Republic until the end of 2023, a total of 727 PWH were registered in HIV clinics across the country as war refugees from Ukraine, amounting to 15–17% of the total number of PWH in care in the country. Within this population, 85.6% were aware of their HIV status and were on stable and suppressive ART. Also, the majority of patients are female (69.5%) with HIV viral load undetectable (< 20 copies/ml) in 79.3% of cases [40]. However, the number of newly HIV diagnosed people previously unaware of their infection is increasing since the beginning of 2022. The proportion of late-presenters among persons with new HIV diagnosis exceeds 50%, with common AIDS-defining conditions (pneumocystosis, TB, and others; https://szu.cz/aktuality/uroven-infekce-hiv-aids-zustava-v-cr-nizka/). The proportion of non-B subtypes has also increased with patients coming from Ukraine, mostly with subtype A, as a major non-B variant. Frequency of transmitted drug resistance is comparable with Czech PWH, but the prevalence of integrase resistance in pretreated persons is higher than typically observed. The initial strategy for maintaining people on ART among already treated cases, including people receiving TLD, was not to change the regimen especially to agents not available in Ukraine to maintain stability of the ART. But due to the fact of unavailability of TLD in fixed single-dose combination, all components of this therapy were given separately. The majority (69.7%) of PWH from Ukraine entering care did not switch therapy following arrival to the Czech Republic, but there was an option for treatment optimization among patients declaring long-term care entry. Hepatitis C treatment with directly acting antivirals (DAA) was provided as needed within the healthcare system. Furthermore, overall strategy and access to comorbidity prevention and screening equal to that of Czech citizens.

Main challenges remain from the beginning of the war: persons with undiagnosed HIV infections with high proportion of late-presenters, including AIDS cases, language barriers, cultural differences, different structure of HIV population, and treatment characteristics, including lack of access to full ART history or unknown resistance profile and different subtype spectrum. The increase in the numbers of children with HIV in need of care is also of challenge.

To cover all these challenges, specific interventions to adjust the care to this new situation had to be made. First, targeted changes were made in the testing strategies reflecting sociocultural specifics of the population originating from Ukraine. This action is performed mainly in collaboration with specialized medical-counseling points and general practitioners, where the numbers of refugees from Ukraine are highest. Differences in epidemiology, vaccination history, and disease awareness were analyzed and preventive activities including HIV, TB, and hepatitis testing applied.

Changes in HIV follow-up care are already ongoing, focusing on translation and understanding, and reflecting specifics based on different HIV-subtypes and differences in resistance profile. The Czech Republic has already extended availability of ART used in pediatric age groups.

### Romania

After the outbreak of the war in Ukraine, the Health Ministry of Romania issued a public document that confirmed public health insurance and free ART for all war refugees. Since March 2022, 156 PWH migrating from Ukraine were registered in Romania. The median age was 38 years (range 5–71) and 62% (97/156) were women. The majority of PWH from Ukraine (78%) were virologically suppressed, and the median CD4^+^ T cell count was 462 cells/μl. Upon arrival, 84% received a DTG-based regimen. Similarly to other EU/EEA countries, the single-tablet combination of TLD is not available in Romania. Overall, 32% of patients switched therapy, in large part due to drug availability. All patients received same day delivery of ART upon doctor's visit, but in the majority of cases for just 30 days [41].

The patients were evaluated initially with routine blood tests, CD4^+^ T cell count, viral load, and serology for hepatitis B, C, and syphilis and as follow-up every 6 months. In total, 6.4% of patients tested positive for hepatitis C, but most of them (4.8%) had previously received treatment for HCV in Ukraine; for the remaining patients, although DAA treatment is free of charge in Romania, they did not receive it, as they are a highly mobile population moving in and out of Romania (i.e., truck-drivers). A symptom-based assessment was performed for all patients to rule-out TB. Two patients (1.2%) had active pulmonary TB, one of which had MDR TB. To date, the number of newly diagnosed HIV cases in this population was low (two diagnoses in people in self-identified as MSM). More than half of the refugees were lost to follow-up, as they were either just transiting Romania to another destination in Europe or have returned to their HIV clinics in Ukraine.

As seen in other EU/EEA countries, the difficulties faced while working with refugees were language barriers and the lack of medical documentation confirming HIV infection or any other diagnosis. Managing long-term care such as chronic diseases or reproductive health will be a future challenge that remains to be addressed.

### Republic of Moldova

It is estimated that more than one million war refugees from Ukraine have transited through the Republic of Moldova. About 100 000–120 000 refugees are currently hosted by the country, but this number is constantly changing [1]. Among those, only 2000 received Moldovan citizenship. This is also reflected in the patterns of migration of refugees with HIV. There are no Ukrainian refugees on HIV treatment who would remain permanently in Moldovian HIV care. In total, 281 patients received ART including five pregnant women and six children. Refugees are accommodated in specially prepared refugee centres or in private homes of Moldovan citizens, living costs being partially covered by international organizations and the state. Socially significant diseases (HIV, TB), as well as health emergencies are covered by the universal medical insurance fund. For other health conditions, insurance can be either purchased, if a residence permit is obtained or requested as part of temporary protection for refugees.

HIV testing of Ukrainian refugees takes place according to the existing algorithm for citizens of the Republic of Moldova, including in risk groups. Twelve patients were newly diagnosed with HIV. Access to HIV care and ART for refugees is available under the same conditions as for country citizens and ART is provided according to the current National Clinical Protocol of the Republic of Moldova with full access to TLD. Treatment efficacy (% with suppressed viral load) is not reported for refugees.

Testing for hepatitis and TB, as well as access to treatment, is available to Ukrainian refugees as for citizens of the Republic of Moldova.

The biggest and ongoing challenge in care is no data on recent tests, past illnesses, or history of ART treatment.

## Summary of achievements and challenges related to provision of HIV care for Ukrainian refugee populations across European countries

As outlined in the country examples provided above, basically from the onset of Russia's invasion in Ukraine, a number of European countries have taken measures to maintain the provision of care among Ukrainian refugees with HIV. Summary of key issues and barriers is outlined in Table 2.

**Table 2:** Key integration issues for Ukrainian migrants in the host country healthcare systems.

Country	Provision of healthcare insurance	Access to general healthcare including primary healthcare	Access to HIV treatment	Access to HCV treatment	Availability of PreP	Availability of anti-TB treatment including against MDR-TB	Key barriers in healthcare provision
Poland	Universal insurance for all Ukrainian migrants who entered country after February 2022, limited for unregistered migrants	Equal to national citizens	Equal to national citizens, immediate access, full state-funded coverage of ART.	Equal to national citizens, local waiting lists.	No state-funded pre-exposure prophylaxis medication available neither to national citizens nor migrants, only purchased generic option possible.	Available free of charge to Ukrainian migrants via dedicated WHO temporary access	Language barrier, lack of pre-exposure prophylaxis, poor access to gender sensitive care (e.g., obs/gyn)
Germany	Universal insurance equal to national citizens	Equal to national citizens	Equal to national citizens, immediate access, full state-funded coverage of ART.	Equal to national citizens, immediate access, full state-funded coverage of treatment	Equal to national citizens, immediate access, full state-funded coverage of PreP	Full state funded coverage of treatment	Language barrier, lack of targeted information, interanal stigma because of HIV status and OST treatment
Czech Republic	Universal insurance for all Ukrainian who entered and has registered as refugees in country after February 2022, limited for unregistered migrants	Equal to national citizens	Equal to national citizens, immediate access, full state-funded coverage of ART.	Equal to national citizens based on local treatment guidelines, no waiting list, full state-funded coverage of treatment	No state-funded pre-exposure prophylaxis medication available neither to national citizens nor migrants, only purchased generic option possible.	Available free of charge, mandatory therapy covered by state irrespective of administrative status	Language barrier, lack of preventive strategies targeted to migrants, low level of disease awareness
Moldova	Universal insurance equal to national citizens	Equal to national citizens	Equal to national citizens, immediate access, full state-funded coverage of ART.	Full state-funded coverage of treatment	Equal to national citizens, immediate access, full state-funded coverage of PreP	Full state-funded coverage of treatment	There are no barriers.
Romania	Universal insurance equal to national citizens	Equal to national citizens	Equal to national citizens, immediate access, full state funded coverage of ART.	Equal to national citizens based on local treatment guidelines, no waiting list, full state-funded coverage of treatment	No state-funded pre-exposure prophylaxis medication available neither to national citizens nor migrants, only purchased generic option possible. Ongoing pilot program at an HIV Check point in Bucharest (regardless migration status)	Available free of charge through the national TB treatment scheme	Language barriers, lack of medical documentation regarding health issues (not only in relation to HIV care)

HIV suppression rates in the Ukrainian refugee populations who initiated ART in Ukraine remain between 80 and 95%, which is in line with the global targets [14]. However, due to unavailability of the fixed-drug single-tablet combination of TLD in most of the countries, it was necessary to switch antiretroviral medication, usually maintaining the core drug class [14]. Timely diagnosis and reduction of the number of people unaware of their HIV status remains a key challenge, with 70–80% of new cases among Ukrainian refugees diagnosed late.

Language barriers and access to medical records related to coexisting comorbidities and medications was reported as one of the key perceived barriers related to provision of medical care to Ukrainian refugees, both in the general population and among PWH [42]. To address the issue of access to medical documentation from the home country, the standardized protocol for clinical management and medical data-sharing for PWH among refugees from Ukraine was established early in 2022 and is now being implemented across European countries [43]. However, there are still areas requiring further standardization, such as the data exchange in case of further migration across European countries.

The standard for provision of care should also consider the needs for the maintenance of the OAT where several barriers were already identified. The key barriers include poor collaboration between providers and nongovernmental organizations, rigidity of formal OAT provision centres in adapting to coordination, and retention challenges in refugee populations, possibly not offering relevant access to OAT care due to low capacity, language barrier, insufficient funding, or distance [44].

## Conclusion

For optimal provision of HIV care among Ukrainian refugees, the exact needs of the population should be of primary consideration. There is also a continuing need for pan-European efforts for the screening for HIV and other infectious diseases in migrating populations to combat late diagnoses. Among populations declaring long term care entry, efforts should be taken to provide relevant monitoring in accordance with the national policies and healthcare system requirements. This should be based on equity of access to care, cultural and individual needs as well as other relevant issues such as reduction of stigma or overcoming the language barrier. Implementation of contingency planning and information for refugees, is essential and should be incorporated in national preparedness plans [45]. The high number of refugees from Ukraine is likely to further affect the TB incidence, increasing the number of individuals with both latent and active infections with drug-resistant strains calling for targeted testing programs and facilitation of access to tuberculostatic therapy including the treatment of drug-resistant strains.

This population is also in need of comprehensive combined prevention strategies, including access to state-funded pre-exposure prophylaxis, which is often unavailable or with limited access to the Ukrainian population.

As an outcome of this review, several key challenges for the provision of high-quality standards of HIV care for Ukrainian migrants have been identified and need further consideration:

(1) Implementation of universal stigma-free testing programs with the overarching aim for the timely diagnosis of HIV and reduction of the number of late diagnosed cases.
(2) Developing legal frameworks allowing for the continued and uninterrupted access to antiretroviral regimens not licensed in EU/EEA in case of contingency or war.
(3) Expansion of refugee targeted TB testing programs with ensured continued access to the first and second line antituberculosis treatment within national systems.
(4) Provision of vaccination especially against HBV.
(5) Improving diagnosis of HCV and sexually transmitted infections with continued equal and state-funded access to relevant therapeutic options including hepatitis C agents.
(6) Continuing molecular surveillance for drug resistance on changes in molecular epidemiology to maintain optimal virologic susceptibility of drug combinations used.

## Acknowledgements

The article, “Provision of care for people with HIV migrating from Ukraine – Preparing for a long-term response,” was commissioned and funded by the European Centre for Disease Prevention and Control (ECDC) as part of the project on European standards for HIV care in Europe.

### Conflicts of interest

The views and opinions expressed herein are the authors’ own and do not necessarily state or reflect those of ECDC. ECDC is not responsible for the data and information collation and analysis and cannot be held liable for conclusions or opinions drawn.
